# Association of physician malpractice claims rates with admissions for low-risk chest pain

**DOI:** 10.1016/j.ajmo.2023.100041

**Published:** 2023-03-26

**Authors:** James Quinn, Sukyung Chung, David Kim

**Affiliations:** a Department of Emergency Medicine, Stanford University School of Medicine, Palo Alto, CA, United States; b Quantitative Science Unit, Stanford University, Palo Alto, CA, United States; c Department of Emergency Medicine, Stanford University, Stanford, CA, United States

**Keywords:** Chest pain, Defensive medicine, Healthcare costs, Malpractice claims rates

## Abstract

**Background:**

Chest pain accounts for 5% of all emergency department visits and accounts for the highest malpractice payout against emergency physicians. To clarify the impact of defensive medicine, we assessed whether admission rates of low-risk chest pain patients are associated with malpractice claims rates.

**Methods:**

A cross-sectional time-series analysis of state-year level malpractice claims rates, admission rates for low-risk chest pain (LRCP; requiring ED physician discretion), and admission rates for acute myocardial infarction (AMI; requiring minimal physician judgment for admission, used as a control) from 2008 to 2017 was performed. Admission rates were derived from Optum's deidentified Clinformatics Data Mart Database. LRCP visits were defined by primary ICD-9 or ICD-10 codes of 786.5, R07.9, or R07.89; length of stay of 2 or fewer days; and no previous major cardiac diagnosis and AMI visits with ICD-9 or ICD-10 codes 410, I21.3, or I121.9. Malpractice claims rates (MPCRs) were derived from the National Practitioner Database (NPD). The association between state-year level MPCR and admission rates for LRCP and AMI was estimated using state fixed-effects models. Standardized costs were inflation adjusted and are expressed in US dollar rate as of 2019.

**Results:**

There were 40,482,813 ED visits during the 10-year study period, of which 2,275,757 (5.6%) were for chest pain, and 1,163,881 met LRCP criteria. Mean age of LRCP patients was 67.8 years, 60.9% were female, and 16.6% were hospitalized, at a mean cost of $17,120. During the same period, 75,266 (0.2%) visits were for AMI, with 87% admitted. The MPCR by state-year varied widely, from 2.6 to 8.6 claims per 100,000 population. A state fixed-effects model showed that an additional physician malpractice claim per 100,000 population was associated with a 3.66% (95% CI 2.02%–5.30%) increase in the admission rate of LRCP. An analogous model showed no association between MPCR and admission rates for AMI (−1.52%, 95% CI −4.06% to 1.02%).

**Conclusion:**

Higher MPCRs are associated with increased admission rates for LRCP, at substantial cost, which may be attributable to defensive medicine in the ED.

## Introduction

Every year in the United States, approximately 6.5 million patients present to the emergency department (ED) with chest pain. Chest pain is the second most common ED complaint, representing 5% of all visits.^1^^,^^2^ Some presentations of chest pain represent acute coronary syndrome (ACS), and missing this diagnosis is devastating for patients and physicians.^3^^,^^4^ Missed ACS is one of the most common malpractice claims against ED physicians and results in the highest average payout against physicians of any ED complaint, $2.3 million.^5^, ^6^, ^7^, ^8^ Fear of litigation and associated risk-aversion has been postulated as a reason for unnecessary admissions of low-risk patients,^9^ but a clear association between liability climate and defensive medicine has been difficult to establish.^10^^,^^11^

Defensive medicine is defined as the use of excessive and unnecessary healthcare resources to minimize physician liability risk.^12^^,^^13^ Survey studies have suggested that liability concerns influence physician decision-making around chest pain^9^^,^^14^, ^15^, ^16^ and that physician practice and utilization patterns vary based on liability concerns.^17^ However, direct evidence of the impact of the liability environment on healthcare utilization and costs is mixed. In particular, the estimated magnitude and direction of the impact vary depending on how liability risk climate is measured.^18^, ^19^, ^20^ Most studies have used state tort reform as a proxy for liability, with inconsistent results. Radiology utilization was shown to vary between states with and without tort reform. Other studies have failed to show differences in Medicare costs between states with and without tort reform.^18^^,^^21^, ^22^, ^23^ The actual malpractice claims rate (MPCR) may be a better measure of the liability risk climate and of physicians’ perception of this risk. Physicians in states with higher MPCR may be more risk averse.^24^ In previous work, we showed that MPCR was associated with admission rates for lower-risk syncope patients.^25^

Chest pain is an optimal complaint with which to study the association between malpractice risk and physician behavior. Chest pain is common and has numerous risk stratification tools to identify low-risk patients, and with the advent of more sensitive troponin assays, most low-risk patients can be safely discharged.^26^, ^27^, ^28^ Physicians are also keenly aware that up to 2% of ACS in ED chest pain patients is missed and that this is a major source of payouts against physicians.^4^^,^^8^ The decision to discharge low-risk chest pain (LRCP) patients requires physician discretion, and we hypothesize that physicians in a higher liability risk environment may be more risk averse and thus more likely to admit these low-risk patients.^29^ Conversely, we would not expect the malpractice environment to affect admission decisions for acute myocardial infarction (AMI), which involves little discretion on the part of the ED physician. In this study, we investigated the potential impact of defensive medicine on healthcare costs by quantifying the relationship between liability risk, measured by a state's MPCR in a given year, and hospital admission rates of LRCP, using admission of AMI as a natural control.

## Methods

### Patient population

Optum's Clinformatics Data Mart Database is a deidentified database comprised of beneficiaries of commercial and Medicare Advantage health plans. From 2008 to 2017, the database contained approximately 16.5 million patients per year, of which 18% were Medicare beneficiaries. The patient population is broadly reflective of the wider US population.^30^ The database contains deidentified claims data for all its members, including details of ED visits, hospital admissions, procedures, and standardized costs, reflecting average payments by the insurer.

### Cohort definitions, calculation of malpractice claims, and admission rates

**Fig. 1:**
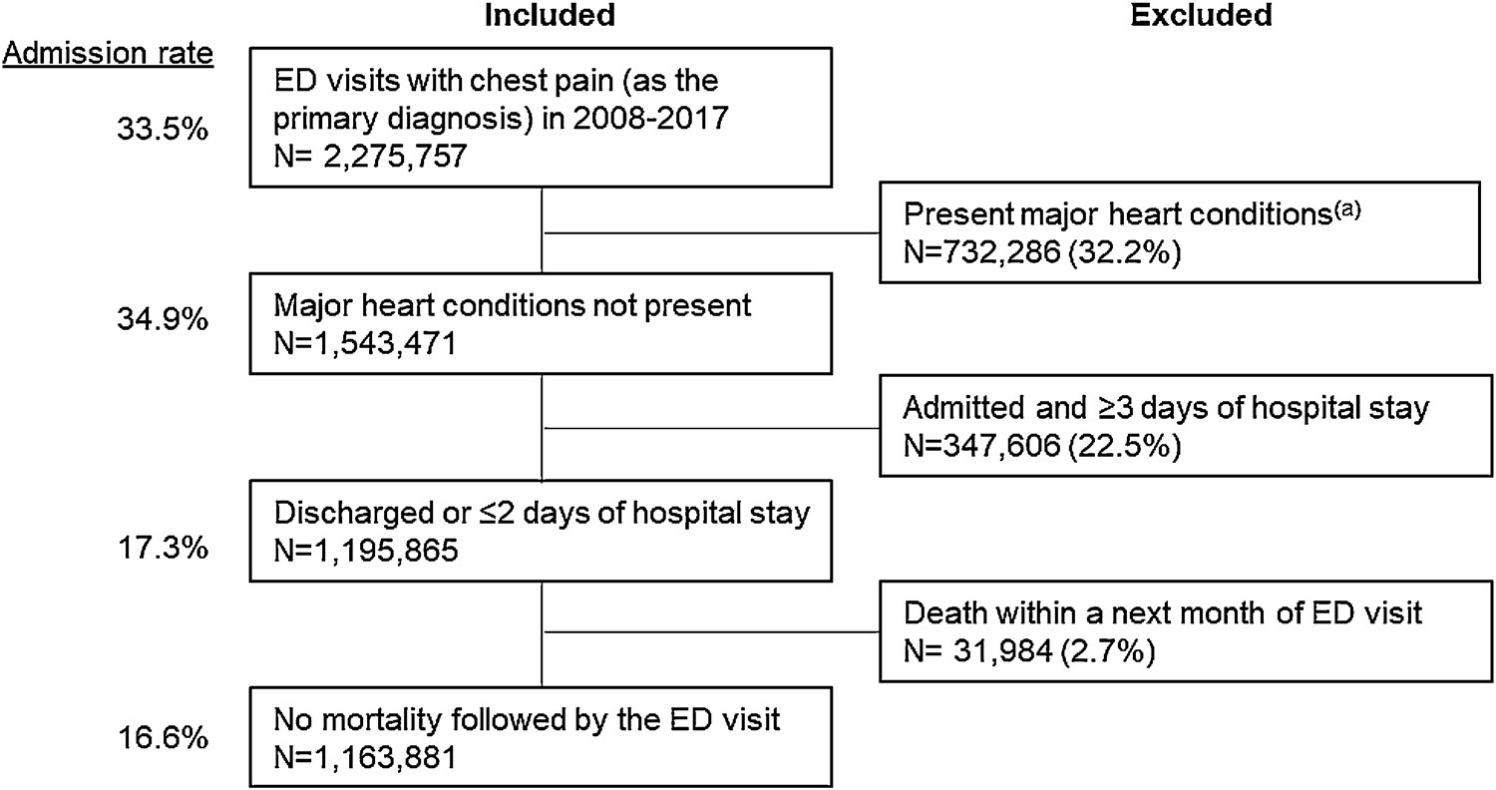
Development of a low-risk chest pain cohort from the Optum data set.

To study the association between the malpractice environment and physician decision-making, we defined an LRCP cohort likely to be discharged after an ED visit (Fig. 1). We included patients with an ED visit with a primary diagnosis code of nonspecific chest pain (ICD-9 786.5, ICD-10 R07.9, R07.89). We excluded patients with a major cardiac diagnosis during or prior to this ED visit (ICD-9 410–414, 425–429, and ICD-10 I20–I25, I42–I51). We excluded patients admitted for 3 or more days, as well as those who died within 30 days of the visit, either of which could indicate a more serious condition meriting admission. We defined admission if the ED visit was associated with hospital confinement, observation stay (CPT 99218–99220, 99224–99226), or inpatient charges of greater than $1000 on day 0, 1, or 2 after the initial ED visit. We calculated the proportion of LRCP visits associated with a hospital admission, in each state and year. We calculated standardized costs associated with each ED visit and subsequent inpatient stay. All costs were inflation adjusted and are expressed in US dollars, rate as of 2019, determined using the Consumer Price Index.^31^ As a control group, for whom admission decisions are generally unambiguous and would not be expected to vary with the malpractice environment, we defined an analogous cohort of ED visits with primary diagnosis of AMI (ICD-9 410 and ICD 10 I42-I51) over the same states and time period.

*% of *N* in the previous criterion.

We used publicly available data from the National Practitioner Database^32^ to calculate state-year level MPCRs. The database contains yearly state-level data on all adverse actions, including malpractice claims, payments, and adverse action reporting against all healthcare providers. When determining the liability risk climate for physicians, we only considered malpractice claims against physicians resulting in payments. To determine the MPCR per 100,000 state population each year, we used the US Census Bureau's population estimates derived from decennial census data combined with estimated birth, death, and migration rates.^33^^,^^34^

The study datasets contained no identifiable human subject data, and the study was deemed exempt from review by the Stanford University Institutional Review Board. This study followed the Strengthening the Reporting of Observational Studies in Epidemiology (STROBE) reporting guidelines.

### Statistical analysis

We compared age, sex, healthcare costs, and associated MPCR between patients who were admitted versus discharged from ED, using two-sided *t*-tests for continuous variables and two-sided chi-squared tests for dichotomous variables.

We estimated state fixed-effects linear models with cluster-robust standard errors, regressing state-year level admission rates for LRCP and AMI on MPCR and controlling for gender and age composition of the population in a given state and year. We applied frequency weights reflecting the number of ED visits per state-year. As state fixed-effect uses only *within-state* variation over time in the estimation, any time-invariant state-level confounders that might affect both admission and MPCR (e.g., unmeasured socioeconomic factors, patient comorbidities, underlying physician practice styles) are eliminated from the model. We used STATA 16.0 for analysis.

## Results

During the 10-year study period, there were 40,482,813 ED visits, of which 2,275,757 (5.6%) were for chest pain, and 1,163,881 visits met criteria for LRCP. Of LRCP patients, 61% were female, and mean age was 67.8 years (standard deviation [SD] [14.0]). Of the LRCP cohort, 16.6% were admitted. Compared to LRCP patients discharged home from the ED, admitted patients were older (mean age of 71.4 [11.1] vs 67.1 [14.4] years), were less likely to be female (53.1% vs 62.5%), presented in state-years with higher MPCR (2.61 [1.44] vs 2.48 [1.41] per 100,000 population), and incurred higher total costs ($20,216 [30,925] vs $3096 [3579]) (Table 1). In the same period, there were 75,266 visits for AMI, with 87% admitted.

**Table 1:** Characteristics of Low-Risk Chest Pain Patients Discharged and Admitted (Unit of observation = ED visit). Mean (SD) for Continuous Variables and *N* (%) for Categorical Variables⁎.

Variable	Overall	Discharged From ED	Admitted to Hospital
	*N* = 1,163,881	*N* = 971,186	*N* = 192,695
Admission	192,695 (16.6%)		
Hospital cost ($), mean (SD)†	5930 (14,475)	3096 (3579)	20,216 (30,925)
ED cost ($), mean (SD)†	2769 (3530)	3096 (3579)	1123 (2729)
Inpatient cost, mean (SD)†	3161 (14,442)		19,093 (30,913)
MPCR, mean (SD)‡	2.50 (1.42)	2.48 (1.41)	2.61 (1.44)
Female, *n* (%)	709,344 (60.9%)	606,942 (62.5%)	102,402 (53.1%)
Age, mean (SD)	67.8 (14.0)	67.1 (14.4)	71.4 (11.1)
18–64, *n* (%)	358,714 (30.8%)	319,666 (32.9%)	39,048 (20.3%)
65–75, *n* (%)	423,924 (36.4%)	347,968 (35.8%)	75,956 (39.4%)
76+, *n* (%)	381,243 (32.8%)	303,552 (31.3%)	77,691 (40.3%)

⁎ For all the variables, differences between discharged and admitted groups were statistically significant (*P* < .001) based on two sample *t*-tests for continuous variables and Pearson's chi-square test for categorical variables.

† In standard cost in US$ at 2019 rate.

‡ Ranges 0.27–8.63 across state-year; *N* = 1,162,892 after excluding 989 ED visits without state information.

**Fig. 2:**
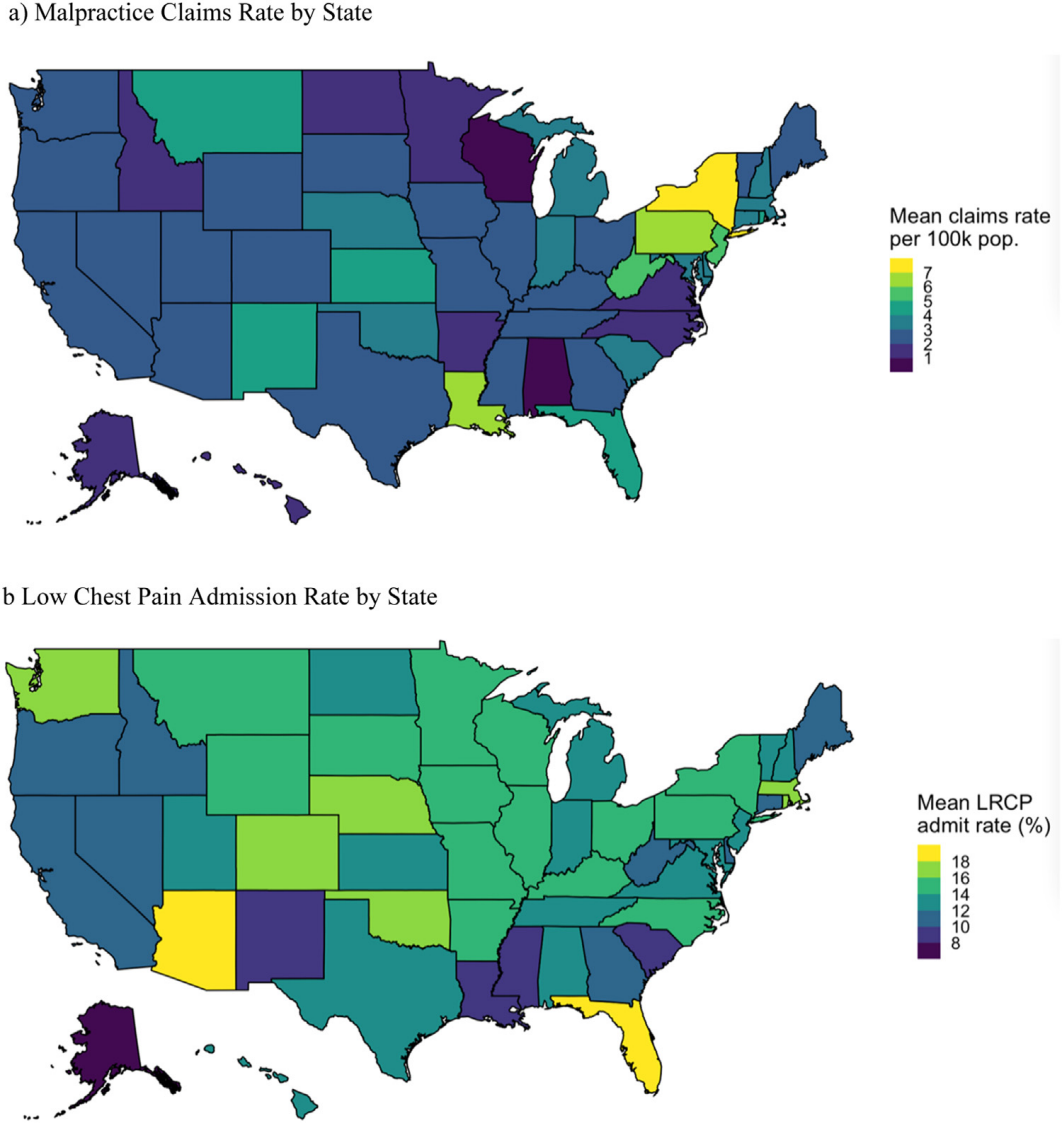
Geographic patterns of MPCR and admissions for low-risk chest pain.

^a^Number of eligible ED visits for each state-year were frequency weighted in the analysis.

**Fig. 3:**
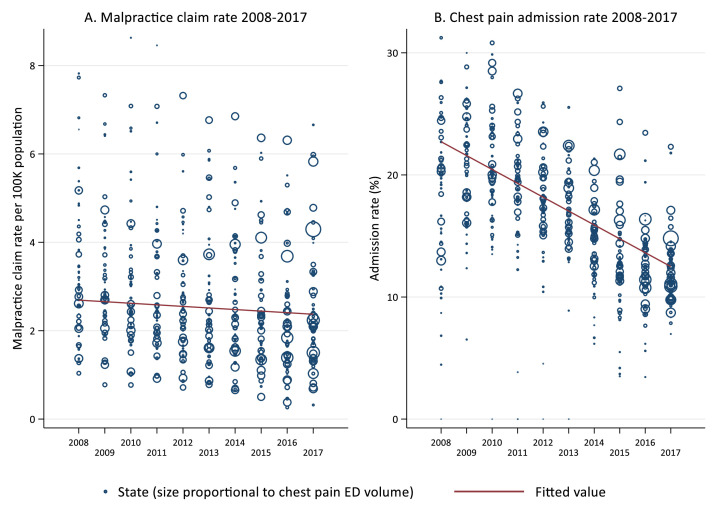
Variation of MPCR and Admission Rates for Low-Risk Chest Pain From 2008 to 2017 (unit: state-year).^⁎^

^⁎^The data were fitted using linear regression (ẞ = −0.035, −1.14 for the left and right graphs, respectively; *P* < .001). Number of eligible ED visits for each state-year were used as frequency weight (proportionate to the size of each circle). States-year with <5 ED visits (*n* = 3) were excluded.

During the 10-year study period, MPCR varied widely across states and years, ranging from 2.6 to 8.6 per 100,000 population (Fig. 2A). Admission rates also varied widely, with an overall declining trend (Fig. 3), (Fig. 2B).

**Fig. 4:**
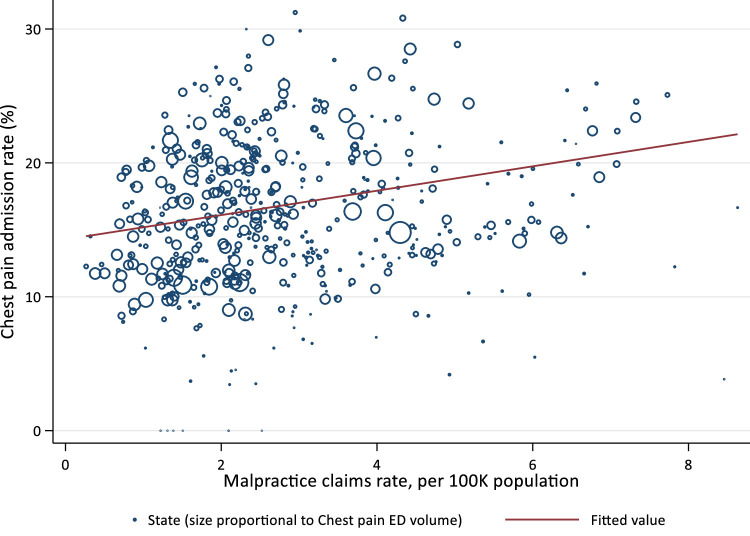
Relationship Between MPCR and Admission Rates for Low-Risk Chest Pain (unit: state-year).^⁎^

^⁎^The data were fitted using linear regression (ẞ = 0.91; *P* < 0.001). Number of eligible ED visits for each state-year were used as frequency weight (proportionate to the size of each circle). States-year with <5 ED visits (*n* = 3) were excluded.

The state fixed-effect model indicated that every 1/100,000 increase in the MPCR is associated with an absolute 3.7% 95%CI (2.02 – 5.30) or a relative 22% increase in the admission rate (Table 2; Fig. 4). For the 75,266 patients with chest pain and AMI (control group), there was no variation of admissions with MPCR −1.52 (95% CI −4.06 to 1.02).

**Table 2:** Malpractice Claims Rate and Low-Risk Chest Pain Admission Rate: Results From State Fixed Effects Regression.

Dependent Variable	Lower-Risk Chest Pain Admission Rate (%)			AMI Admission Rate (%)		
	Coefficient	95% Conf. Interval		Coefficient	95% Conf. Interval	
		Lower	Upper		Lower	Upper
Claims per 100K population)	3.659⁎⁎⁎	2.024	5.295	−1.520	−4.056	1.016
% Female	−0.574⁎⁎⁎	−0.845	−0.302	0.025	−0.110	0.159
Age (reference: % Age 65–75)	0.075	−0.062	0.213	−0.175	−0.378	0.029
% Age 18–64	0.198*	0.006	0.391	−0.133⁎⁎	−0.228	−0.037
% Age ≥ 76	34.146⁎⁎⁎	18.084	50.209	97.041⁎⁎⁎	87.015	107.067
Constant	3.659⁎⁎⁎	2.024	5.295	−1.520	−4.056	1.016
Weighted *N*	1,162,920			75,266		

⁎ *P* < .05.

⁎⁎ *P* < .01.

⁎⁎⁎ *P* < .001.

## Discussion

In this large national cohort, we found that higher state-year level MPCR was associated with higher admission rates of lower-risk patients presenting to the ED with chest pain. The higher admission rates in states and years accounted for an extra $330 million in costs in this study population alone. Given a modest representation of private insurance among the Medicare population (mostly through Medicare Advantage), the potential impact among fee-for-service Medicare enrollees would likely be much larger than the costs reported here. Studying a lower-risk chest pain cohort that is generally discharged but requires physician discretion showcases a compelling association between liability risk and potential defensive medicine as manifest in unnecessary hospital admissions.

The costs of excess hospitalizations we report are impressive, but the true cost of defensive medicine may be higher still.^35^^,^^36^ Defensive medicine may expose patients to unnecessary procedures and admissions, resulting in substantial nonmonetary and indirect costs, in addition to direct costs of unneeded tests and hospitalizations.^24^^,^^37^^,^^38^ The cost of false-positive tests and admissions leading to further tests and unnecessary treatments are well documented, including risks of medical error and hospital-acquired infections.^39^^,^^40^ These harms are difficult to quantify and are not reflected in the costs reported in this study.

In this study, we used actual MPCRs against physicians as a measure of liability risk. Most previous studies of the association between liability risk and utilization have used tort reform rather than MPCR as a proxy for liability climate, with variable results.^21^, ^22^, ^23^ There are several drawbacks to using tort reform as a proxy for physicians’ perception of malpractice risk.^18^ First, tort reform takes many forms, and payment caps of $1–2 million dollars may have little impact on the pursuit of malpractice litigation. Second, changes in malpractice rates may lag policy changes like tort reform. Finally, some states may reverse tort reform policies based on the ruling political party, preventing enduring change in the malpractice climate and in physicians associated behavior.^10^ We believe that the actual MPCR is a better proxy for the liability climate as perceived by physicians and have established this approach in previous work on lower-risk syncope patients.^25^

Hospitalization of lower-risk patients with chest pain is now recognized to have little clinical benefit. With risk stratification tools such as the HEART score and the advent of more sensitive troponin assays, physicians can accurately identify most patients who can be safely discharged with little risk of a major adverse event.^28^^,^^26^ Decision support and society guidelines are tools that can support judgment to offset a “zero miss rate policy” thought to be responsible for defensive medicine. These tools likely have a greater impact on physician behaviors than policy measures like tort reform.^10^ As evidence, we observed a trend toward fewer LRCP admissions over the 10-year study period (Fig. 2B). that corresponds to the advent of clinical decision tools and society guidelines for chest pain. Furthermore, their availability via electronic medical records, online calculators, and smartphone applications has improved their utilization during this time.^41^ The LRCP cohort developed for this study was defined with standardized codes for nonspecific chest pain and excluded patients with serious cardiac diagnoses, prolonged admissions, or death within 30 days of the ED visit. While we were not able to apply exact guideline criteria to this cohort, we are confident that most patients in the LRCP cohort would generally be candidates for discharge based on existing guidelines.

Despite the overall trend toward fewer LRCP admissions, the rate of LRCP admission is still likely greater than clinically necessary and reflects the reality of physician practice in the United States.^42^ There is tremendous variability in physician practice, and higher admission rates have not been associated with better patient outcomes.^43^ Despite risk scores and consensus guidelines, physicians remain risk averse and admit many low-risk patients. In a point-of-care survey of physicians admitting chest pain patients to rule out ACS, physicians reported that they would not have admitted 29% of the patients if the medicolegal environment allowed for a nonzero acceptable miss rate.^17^ There are other reasons physicians may admit low-risk patients, including variation in healthcare systems, physician training, and experience.^44^, ^45^, ^46^ Given the complexity of these interactions, it is not feasible to strictly isolate the contribution of malpractice risk to physician behavior. Nevertheless, this study demonstrates a significant association between the local malpractice environment and admission rates for LRCP, which may reflect the effect of perceived liability risk on physician decision-making around one of the most common and consequential ED complaints.

### Limitations

The Optum Clinformatics Data Mart Database tracks patients with commercial insurance or Medicare advantage and so excludes the uninsured and Medicaid populations. We were not able to directly account for provider factors that may impact physician risk aversion and decision-making, though the inclusion of state-fixed effects mitigates any bias introduced by characteristics of a state's stable physician population. We believe our findings to be robust to practice type (academic, community, rural, member-based system) and physician factors (e.g., experience and training), given the overall representativeness of this large national cohort. We were unable to observe all payments (e.g., settlements) and other burdens of the liability system, such as a physician being named in a claim and having to defend herself, but we believe the claims rates we observe to better represent the local state malpractice environment than state tort reform policies.

Finally, we assembled a lower-risk cohort retrospectively without the availability of important variables such as the ECG characteristics and troponin values commonly used in chest pain risk assessment, though practice standards suggest it is unlikely for patients with evidence of myocardial injury by either measure to be discharged. The purpose of this study was not to retrospectively risk-stratify patients but rather to identify a lower-risk chest pain cohort requiring physician discretion to determine disposition, as well as a control cohort whose disposition would not be expected to be influenced by malpractice risk. This approach allowed us to determine the association between the malpractice environment and a common and consequential physician decision.

## Conclusion

Admission rates for ED patients with lower-risk chest pain are strongly associated with local MPCRs, which may reflect defensive medicine. Physicians are nearly unanimous about the existence of defensive medicine, but its true causes and impacts have been elusive. Physician perceptions of liability risk are one likely driver of behavior, with substantial costs to patients and health systems (Figs. 3 and 4; Table 2).

## Role of funder

Data for this project were accessed using the Stanford Center for Population Health Sciences Data Core. The PHS Data Core is supported by a National Institutes of Health National Center for Advancing Translational Science Clinical and Translational Science Award (UL1 TR001085) and Internal Stanford funding. The contents are solely the authors’ responsibility and do not necessarily represent the official views of the NIH.

## Declaration of Competing Interest

None of the authors have any financial or personal relationships with other people or organizations that could inappropriately influence our work.
